# Preparation of W-C-Co Composite Micropowder with Spherical Shaped Particles Using Plasma Technologies

**DOI:** 10.3390/ma14154258

**Published:** 2021-07-30

**Authors:** Andrey Samokhin, Nikolay Alekseev, Aleksey Astashov, Aleksey Dorofeev, Andrey Fadeev, Mikhail Sinayskiy, Yulian Kalashnikov

**Affiliations:** Baikov Institute of Metallurgy and Materials Science of the Russian Academy of Sciences, 49, Leninskiy prosp., 119334 Moscow, Russia; nvalexeev@yandex.ru (N.A.); aastashov@imet.ac.ru (A.A.); adorofeev@imet.ac.ru (A.D.); afadeev@imet.ac.ru (A.F.); sinaisky@imet.ac.ru (M.S.); ulian1996@inbox.ru (Y.K.)

**Keywords:** tungsten carbides, cobalt, nanopowder, synthesis, granulation, spheroidization, DC thermal plasma

## Abstract

The possibility of obtaining composite micropowders of the W-C-Co system with a spherical particle shape having a submicron/nanoscale internal structure was experimentally confirmed. In the course of work carried out, W-C-Co system nanopowders with the average particle size of approximately 50 nm were produced by plasma-chemical synthesis. This method resulted in the uniform distribution of W, Co and C among the nanoparticles of the powder in the nanometer scale range. Dense microgranules with an average size of 40 microns were obtained from the nanopowders by spray drying. The spherical micropowders with an average particle size of 20 microns were received as a result of plasma treatment of 25.36 microns microgranule fraction. The spherical particles obtained in the experiments had a predominantly dense microstructure and had no internal cavities. The influence of plasma treatment process parameters on dispersity, phase, and chemical composition of spherical micropowders and powder particles microstructure has been established.

## 1. Introduction

Hard metals based on tungsten carbide are widely used in the production of cutting tools for metalworking, tools for rock drilling, wear-resistant parts, and coatings, etc. [1,2,3].

Hard metal products are manufactured by various methods, including powder metallurgy methods, but the production of complex-shaped parts encounters significant difficulties. Nowadays, intensively developing additive technologies make it possible to overcome the problems of manufacturing parts with complex shapes, so the attention of researchers and developers is drawn to the development of additive technologies for the manufacture of hard metal compacts. An overview of current research in this area can be found in [4].

Metal powders used in the layer-by-layer growth of products by methods of additive technologies should have good flowability and provide the highest possible packing density of particles when creating a powder layer [5,6]. These requirements may be achieved by using powders with a spherical particle shape having a size in the range from 20 to 60 μm.

An effective method for producing powders with a spherical particle shape is the processing of powders consisting of particles with an irregular shape in a flow of thermal plasma of electric discharges where the particles melt and become spherical due to surface tension forces. Plasma processes of spheroidization, the studies of which were started in the second half of the last century [7,8], are now widely used for processing metal powders [9,10,11,12,13,14]. However, the production of powders of WC-Co hard metals with a spherical particle shape in plasma processes has been studied only in a very limited number of works.

The authors of [15] obtained spherical powders by processing composite microgranules WC-12 wt.% Co in a thermal plasma flow generated by an electric arc plasma torch. The initial microgranules had an average size of 38 μm and were obtained by spray drying a suspension of WC and Co powders. Plasma treatment of microgranules made it possible to reduce the porosity in composite microparticles and ensure their spheroidization; however, high-temperature treatment led to undesirable effects—the transformation of some parts of WC carbide into W_2_C and the partial evaporation of cobalt. The size of WC grains in the obtained spherical particles was in the micron range.

The authors of the paper [16] showed the possibility of obtaining dense spherical microparticles of the hard alloy WC-Co as a result of processing the corresponding porous microgranules in a thermal plasma flow followed by a heat treatment. Initial WC-30 wt.% Co microgranules with an average size of 87 μm were obtained by spray drying a suspension of a mixture of initial WC and Co powders. As a result of the processing of porous microgranules, dense spherical particles with a changed phase composition, represented by the phases C, Co, W_2_C, and Co_3_W_3_C, were obtained. Subsequent heat treatment of this powder at a temperature of 950 °C provided a return to the original phase composition WC-Co, while the particles retained their spherical shape, and the powder had the fluidity required for its use in additive technologies.

In the above-mentioned works [15,16], the obtained spherical WC-Co particles had a microstructure with micron-sized WC grains.

The particle size of tungsten carbide powder is one of the critical factors determining the mechanical properties of WC-Co hard metals and the transition to nanostructured hard metals is a means to significantly improve these properties [17,18].

The aim of this research was to experimentally determine the possibility of obtaining composite W-C-Co system micropowders with a spherical particle shape having a submicron/nanoscale internal structure, using an approach involving three successive stages:

- W-C-Co system nanopowders synthesis by interaction of tungsten oxide WO_3_ and cobalt powder mixture with methane in a flow of hydrogen-containing thermal plasma of electric arc plasm torch.
- Nanopowders granulation in a spray dryer to produce nanopowder microgranules, and classification of microgranules into a given fraction.
- Densification and spheroidization in a thermal plasma of the separated fractions of microgranules.

In some cases, the final classification of dense spherical particles obtained after plasma treatment is required with the purpose of removing nano- and submicron particles formed during condensation of vaporized material.

The proposed approach can make it possible to obtain micropowders of a WC-Co hard metal with a spherical particle shape and a submicron microstructure. To date, such powders have not been produced, but they are of interest for the manufacture of hard metal parts of complex shapes with an ultrafine microstructure using modern methods of additive technologies.

## 2. Materials and Methods

### 2.1. Obtaining Nanopowder of W-C-Co System

W-C-Co system nanopowder was obtained by processing of mixture of tungsten oxide WO_3_ and cobalt Co powders with methane in thermal plasma flow generated in electric arc plasma torch with self-adjusting arc length with nominal power of 30 kW. Detailed description of the setup and processes of nanoparticles formation in the thermal plasma flow are presented in [9].

Powder mixture consisted of tungsten oxide WO_3_ particles with average particle size less than 50 µm and Co particles with particle size less than 5 µm. All experiments were performed at a constant mass ratio of elements W/Co = 11 in the raw mix.

Nitrogen, as a part of plasma-forming and transport gases, was supplied from the air separation unit, the oxygen content in nitrogen was no more than 0.01 vol.%. Hydrogen, with purity not less than 99.95 vol.%, was added to plasma-forming gas to ensure reduction of tungsten oxide. Compressed methane, with a purity of 99.9 vol.%, was used as a carbidizer.

### 2.2. W-C-Co System Nanopowder Microgranules Production

Nanopowder microgranules of W-C-Co system were obtained by spray drying of aqueous suspensions of W-C-Co nanopowder on Buchi Mini Spray Dryer B-290 (Flawil, Switzerland) equipped with an ultrasonic nozzle.

The production of microgranules of the W-C-Co system based on spray drying includes three main stages:

- Preparation of an alcohol suspension consisting of composite nanoparticles of the W-C-Co system and polyvinyl butyral (C_8_H_14_O_2_)_n_ (PVB) used as an organic binder to ensure the strength of the obtained microgranules.
- Spray drying of the obtained suspension with an ultrasonic nozzle. Nitrogen was used as a working gas in the process of granulating the nanopowder using a Buchi B-295 circulation gas circuit.
- Separation of the 25.63 μm fraction of microgranules on a Retsch AS 200 sieve machine (Haan, Germany).

### 2.3. Plasma Processing of W-C-Co Nanopowders

Separated fraction of nanopowder granules was treated in a thermal argon-hydrogen (3 vol.%) plasma jet, generated in an electric arc plasma torch with a rated power of 30 kW. A detailed description of the plasma setup for powders spheroidization used is given in [10]. The formation of nano- and submicron particles formed due to partial evaporation and subsequent condensation of a raw material. Destruction of some granules by collision and removal of thermal gasification products of an organic binder from granules enhances the evaporation processes during plasma treatment of nanopowder microgranules. Sedimentation in a distilled water after ultrasonic dispersion was used to remove nano- and submicron particles from plasma treated powder. In this process, spheroidized microparticles are separated from the resulting suspension by sedimentation and subsequent drying in vacuum, while nano- and submicron particles are removed with water as a suspension.

A comprehensive analysis of the physicochemical properties of powders included:

- Scanning, transmission electron and optical microscopy—Scios SEM microscope (FEI, Hillsboro, OR, USA) with elemental energy dispersive X-ray microanalysis (EDS), Osiris TEM microscope (FEI, Hillsboro, OR, USA) and Olympus CX31 OM microscope (Tokyo, Japan), respectively. ImageScope M software (Aperio Technologies, Vista, CA, USA) was used for statistical image processing.
- Measurement of the specific surface area of nanopowders by the BET method on a Micromeritics TriStar 3000 specific surface analyzer (Norcross, GA USA).
- Amount of total oxygen measurements with a TC-600 (LECO, St. Joseph, MO, USA) analyzer during reduction smelting in a graphite crucible and detection of the resulting gases with an infrared radiation sensor.
- Amount of total carbon in a CS-600 (LECO, St. Joseph, MO, USA) analyzer by burning the sample in a stream of oxygen and detecting the resulting gases using an infrared radiation sensor.
- Determination of metallic elements (Co, W) by X-ray fluorescence spectroscopy (XRFMS) in the powder layer on an Orbis analyzer (EDAX, Mahwah, NJ, USA).
- Particle size distribution of micropowders measured with a Mastersizer 2000M laser diffraction particle size analyzer (Malvern, Worcestershire, UK) with Hydro S liquid sample feeder.
- Phase analysis on an Ultima-4 X-ray diffractometer (RIGAKU, Tokyo, Japan) with a monochromator in filtered Cu-Kα radiation.
- Separation of nanoparticles in spheroidal micropowders by fractional separation in liquid by sedimentation of aqueous suspension after treatment on ultrasonic dispersant Bandelin Sonopuls HD3100 (Berlin, Germany).
- Determination of flowability of powders was carried out using a calibrated funnel (Hall device) and a stopwatch for samples weighing 50 g.
- Determination of the apparent density of powders by weight method using a funnel in accordance with GOST 19440-94 [19] (ISO 3923-1:2018-09 [20], ISO 3923-2:1981 [21]).

## 3. Results and Discussion

### 3.1. Preparation of W-C-Co System Nanopowders

Plasma-chemical synthesis of W-C-Co system nanopowders was carried out in the plasma reactor with the confined jet flow. The power of the plasma torch was in the range of 19.24 kW. A mixture of nitrogen and hydrogen (20 vol.%) was used as plasma-forming gas. Dispersed raw material was transported with a feeding rate of 5 g/min by a hydrogen–methane mixture.

**Figure 1:**
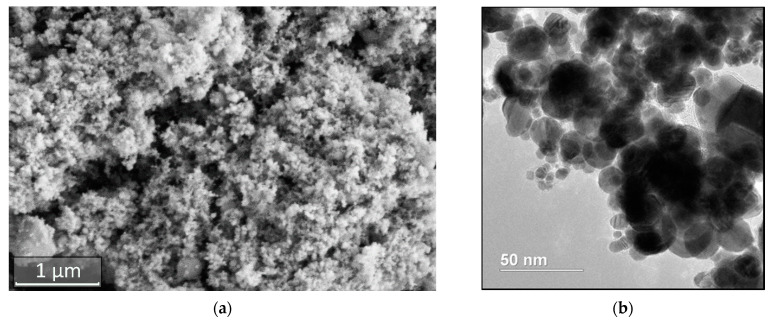
SEM (**a**) и TEM (**b**) images of W-C-Co nanopowder.

According to the results of electron microscopy (Figure 1), obtained nanopowders represent a polydisperse system consisting of aggregated nanoparticles in the size range from 10 to 100 nm, with the nanoparticles predominantly close to spherical in shape.

The specific surface area of nanopowders obtained ranged from 15 to 20 m^2^/g. The main process parameters affecting the values of the specific surface area are the feed rate of dispersed raw materials and the methane flow rate. With increasing feed rate of dispersed raw materials, the specific surface area decreases due to a boost in the concentration of condensed vapors, and an increase in methane flow rate leads to a boost in the specific surface area of the nanopowder due to an increase in the content of free carbon with a high specific surface area.

**Figure 2:**
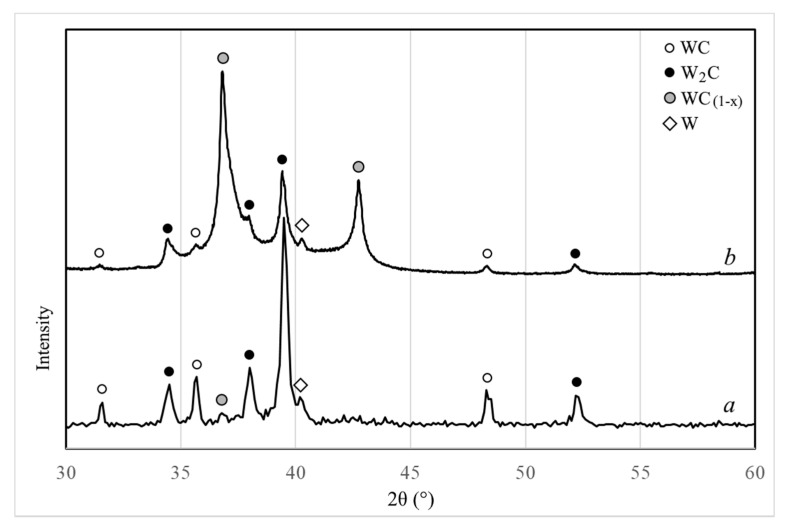
XRD patterns of W-C-Co (**a**) and W-C (**b**) nanopowders produced by plasma synthesis.

Phase composition of the obtained W-C-Co system nanopowder containing 4.7 wt.% of carbon is characterized by the predominance of the W_2_C tungsten carbide phase with the presence of WC and W phases (Figure 2a).

It is noted that during the plasma-chemical synthesis of nanopowders of the W-C and W-C-Co systems, the phase composition of the resulting nanopowders is noticeably different (Figure 2). Introduction of a cobalt into the process leads to a significant decrease in a cubic tungsten carbide phase WC_1-x_ and an increase in hexagonal tungsten monocarbide phase WC content as well as an increase in W_2_C phase.

**Figure 3:**
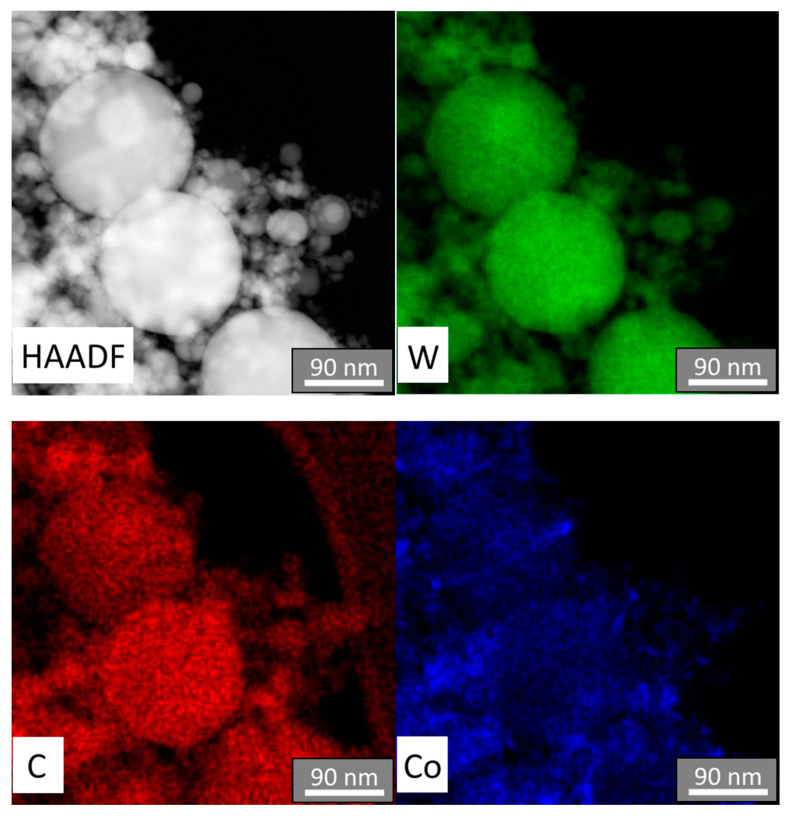
Results of the EDS microanalysis with element distribution maps (W, C, Co) among particles of W-C-Co composite nanopowder.

The results of the EDS microanalysis indicate that the elements W, Co, and C are evenly distributed among the nanoparticles of the powder of the W-C-Co composition with a uniformity scale in the nanometer size range (Figure 3). The high level of uniformity in the elemental composition of the obtained nanopowder is determined by the mechanism of its formation based on the co-condensation of components from the gas phase. Therefore, when carrying out this process, special attention was paid to creating structural and technological conditions to ensure the most complete evaporation of the initial disperse raw materials and the implementation of the targeted chemical reactions. The yield of the W-C-Co composition nanopowder reached 98.0–99.5 wt.%. The content of micron-sized particles in the nanopowders did not exceed 0.5–1.5 wt.%. We attribute their content mainly to incomplete evaporation of the raw material particles. The oxygen content in this fraction was 3–5 wt.%.

For the following stages of nanopowder granulation and microgranule spheroidization, a batch of W-C-Co system nanopowder containing 7.7 wt.% cobalt and 4.7 wt.% carbon was produced. Total oxygen content of the powder was 0.5 wt.%. The carbon content in the nanopowder was reduced in relation to the value corresponding to tungsten monocarbide WC since an organic binder was used for making microgranules which pyrolysis during plasma treatment leading to the formation of carbon in the microgranule volume.

### 3.2. Preparation of W-C-Co microgranules

The granulation process of the obtained nanopowders was carried out in a spray drying unit at drying gas temperatures of 40–150 °C with a flow rate of 20 m^3^/h. The nozzle cooling gas flow rate was 0.3 m^3^/h. Flow rate of the suspension was in the range from 3 to 12 g/min. The power of the ultrasonic nozzle in all cases was 3 W.

**Figure 4:**
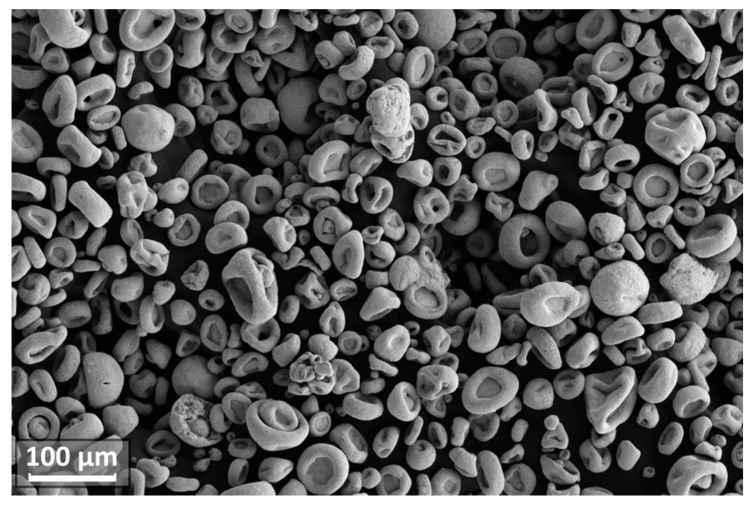
SEM image of W-C-Co microgranules obtained in the process of spray drying.

The microgranules obtained in the spray drying process were subjected to sieving with the extraction of fractions of 25–63 microns, the yield of which was about 50%. Microgranules have mostly irregular shapes (Figure 4), determined by drying conditions, as well as the use of a low-boiling liquid-ethanol—as a dispersion medium. When distilled water and water-soluble organic binder were used as a dispersion medium we obtained spherical granules, but their strength was insufficient, and the yield of the target fraction was low. This is largely due to the fact that nanopowders of the W-C-Co system have a pyrocarbon layer on the surface that is formed as a result of hydrocarbons pyrolysis [22] and, therefore, the water suspensions of the W-C-Co nanopowder system have a poor stability.

**Figure 5:**
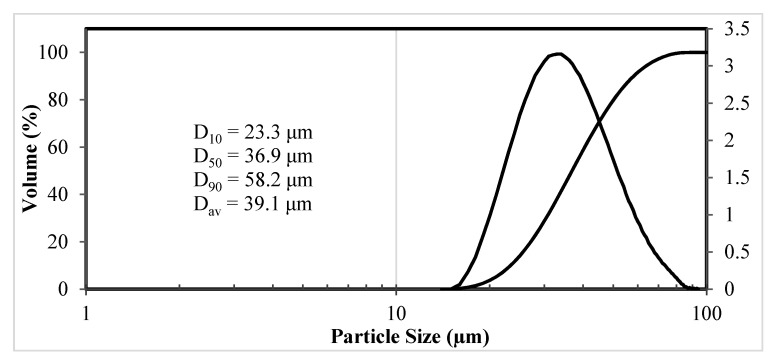
Differential and integral microgranules size.

The dispersed composition of separated granule fraction was characterized by the values of distribution parameters D_10_ = 23 μm, D_50_ = 37 μm, and D_90_ = 60 μm (Figure 5). The average particle size of microgranules was 39 μm.

According to the analysis results, the carbon content in the microgranules was 6.4 wt.%, oxygen-1.0 wt.%, the apparent density of the microgranules was 2.6 g/cm^3^, and the flowability was 29 s/50 g. An increase in carbon content by 1.7 wt.% and oxygen content by 0.5 wt.% was due to the use of an organic binder. An additional channel for increasing oxygen in the granules was the interaction of nanoparticles with active oxygen-containing components formed during ultrasonic dispersion of an alcohol-based suspension at the stage of preparing a suspension for spray drying.

Selected granulation mode in combination with the use of an alcohol-based dispersion medium and polyvinyl butyral made it possible to ensure the strength of microgranules sufficient for their transportation without destruction from the powder feeder into the thermal plasma flow by the carrier gas.

### 3.3. Plasma Treatment of W-C-Co System Microgranules

Fraction of nanopowders with a size range of 25 to 63 microns containing 6.4 wt.% carbon was treated in a thermal plasma jet with a mixture of Ar + 5 vol.% H_2_. Use of hydrogen in the plasma-forming gas allowed to increase the intensity of heating of granules due to its high thermal conductivity and, as a result, the degree of spheroidization of the obtained particles. At a plasma-forming gas flow rate of 2.0 m^3^/h, the power input of the plasma torch in the conducted experiments was 20–30 kW. The enthalpy of the plasma jet varied in the range from 2.4 to 4.9 kW∙h/m^3^. Microgranules with a flow rate of 6 g/min were transported from the feeder to the plasma reactor with argon at a flow rate of 0.5 m^3^/h.

**Figure 6:**
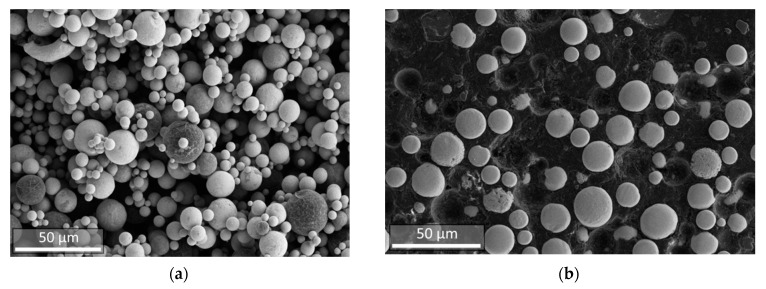
SEM images of W-C-Co micropowder after the spheroidization and removal of nano- and submicron particles (**a**) and metallographic cross-section of this powder (**b**).

In all experiments performed, within the specified range of process parameters variation, the degree of spheroidization of microgranules was at least 90% (Figure 6a). Apparent density of obtained spheroidized powder (after removal of nano- and submicron particles) changed from 8.8 to 9.6 g/cm^3^ with an increasing enthalpy of the plasma jet (Table 1).

The experiments performed showed that an increase in the enthalpy of the plasma jet in the range from 2.4 to 4.9 kW h/m^3^ leads to nanoparticles content increasing in the treated powder from 8 to 13 wt.%. The formation of nanoparticles occurs due to the evaporation and subsequent condensation of microgranule components. The elemental composition of the nanoparticles is characterized by a cobalt content of 70 wt.% and a tungsten content of 30 wt.%. The predominant content of cobalt in nanoparticles relates to a lower boiling point of this metal and, as a consequence, its intensive evaporation from the surface of spheroidized granules in plasma. The presence of tungsten in nanoparticles may be caused by the removal of tungsten carbide nanoparticles from the surface of microparticles by the cobalt vapor during evaporation, as well as by the destruction of nanopowder microgranules during thermal decomposition of the organic binder with an active gas release.

**Figure 7:**
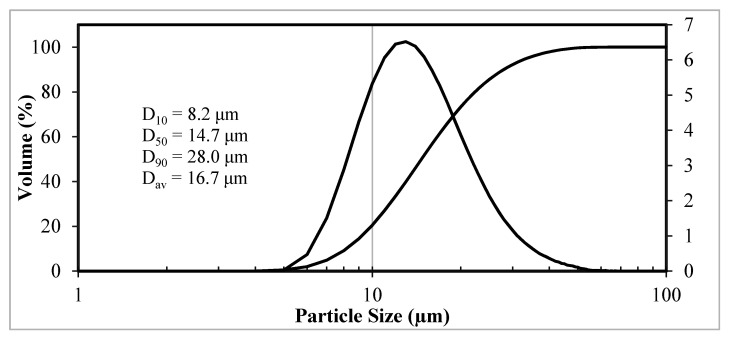
Differential and integral particle size distribution of W-C-Co micropowder after treatment in plasma and removal of nano- and submicron particles.

The particle size distribution in the spheroidized powder after removal of nano- and submicron particles formed is shown in Figure 7. The disperse composition of the spheroidized powder is characterized by values D_10_ = 8 microns, D_50_ = 15 microns, and D_90_ = 28 microns. Compared with initial microgranules, the spherical particles are smaller (Figure 5 and Figure 7), which may be related not only to the acquisition of a more compact spherical shape of particles but also to the destruction of microgranules in the plasma flow as a result of gas release at thermal decomposition of the organic binding used at granulation.

Thermal plasma jets generated by electric arc plasma torches are characterized by significant enthalpy and gas velocity gradients. In the plasma jet processing of polydisperse powders, the conditions of thermal interaction of particles of the processed material with the high-temperature gas are different. This fact predetermines possible differences in directions and rates of phase transformations occurring in individual particles, so that, under those conditions, particles in polydisperse powders may have different internal microstructures.

**Figure 8:**
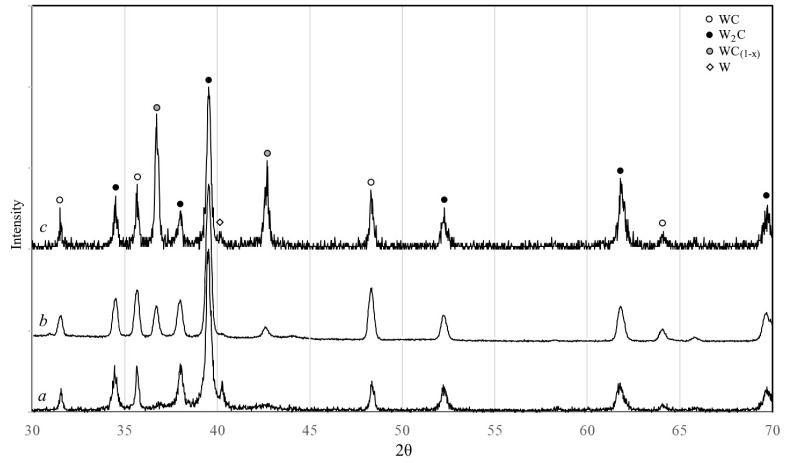
XRD patterns of initial W-C-Co nanopowder (**a**) and W-C-Co powder spheroidized at different plasma enthalpy: (**b**) 2.8 kW∙h/m^3^, (**c**) 4.8 kW∙h/m^3^.

The change in phase composition in the process of nanopowder transformation into spherical microparticles is presented in Figure 8. The main phase in both nanopowder and spherical microparticles remains the tungsten carbide phase W_2_C. When processing granules in a plasma flow the carbide phase WC_(1−x)_ formation occurs and its content considerably increases with an increase in plasma stream enthalpy: by the results of XRD relative intensity of 100% reflections increases from 0.3 to 0.85. The content of tungsten monocarbide WC, obviously, also increases, as the ratio of relative intensity of 100% reflections WC and W_2_C increases from 0.28 to 0.43. Increasing the enthalpy of the plasma jet during microgranules processing leads to an increase in the average mass temperature of the gas-dispersed stream and the microparticles present in it, which, in turn, may contribute to a rise in the content of the higher-temperature carbide phase WC_(1−x)_.

In addition to the plasma enthalpy, there are several other factors that should significantly influence the phase composition of the W-C-Co spheroidized powder. These include the total carbon content of the nanopowder, the value of the specific surface area of the nanopowder, and the residence time of nanopowder granules in the high temperature region during plasma spheroidization. Within the framework of this work, the study of the influence of these factors was not carried out since the main task was to demonstrate the fundamental possibility of implementing the process of obtaining dense spherical microparticles of a composition based on tungsten carbides and cobalt with a submicron microstructure. A separate study is also required to determine the change in the phase composition and the structure of the resulting material in the L-PBF process using spheroidized micropowder of the W-C-Co system.

**Figure 9:**
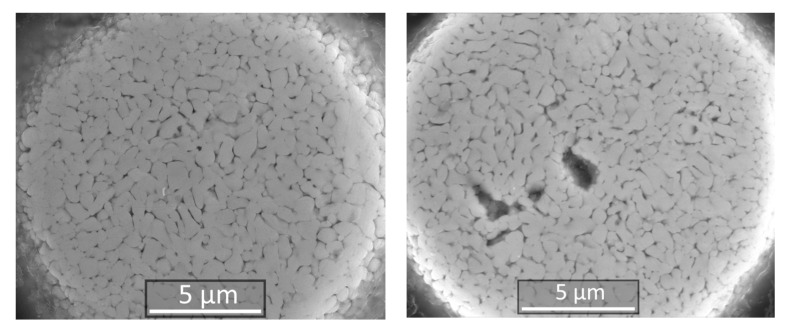
SEM images of W-C-Co spheroidized micropowder cross-section.

Spherical particles obtained in the experiments had predominantly dense microstructure and had no internal cavities, although some particles had small pores (Figure 6b and Figure 9). Grain size of the particles in most cases was in the submicron range.

**Figure 10:**
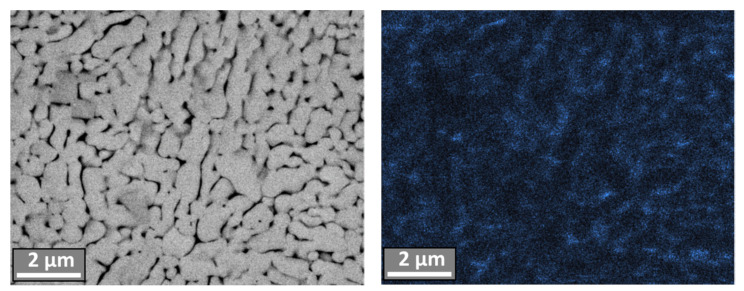
SEM image of W-C-Co spheroidized micropowder cross-section and map of Co distribution in this cross-section.

According to the EDS results of individual micropowder particles cross-sections with the most characteristic morphology revealed that the cobalt in them is uniformly distributed at submicron level in accordance with the structure of the particle (Figure 10).

**Figure 11:**
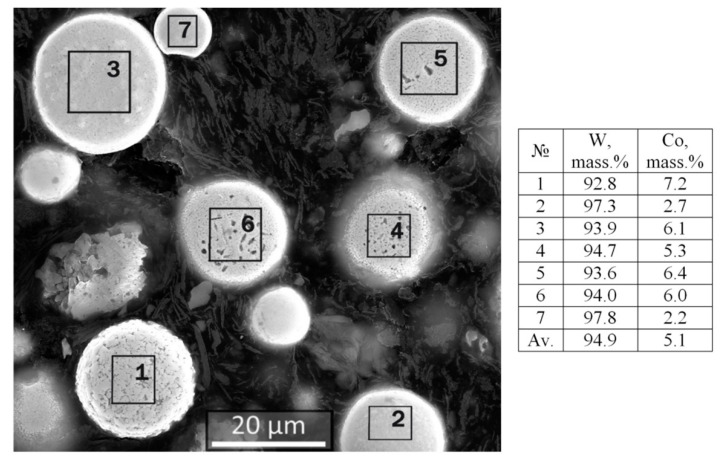
SEM image of W-C-Co micropowder cross-section and the results of EDS elemental microanalysis showing the Co and W content in the powder particles with different structures.

As a result of elemental microanalysis of a cross-sectional area on ground spheroidal particles of W-C-Co composition (Figure 11), it is found that the amount of cobalt in particles of different size and structure varies in a wide range from 2.2 to 7.2 wt.% and averages about 5 wt.% for W+Co calculation (without carbon), which is approximately 4.7 wt.% for the W-Co system.

Intense evaporation of cobalt in the process of plasma spheroidization of granules led to a noticeable decrease in its total content in the obtained spherical powder. In initial microgranules the cobalt content (when analyzed by the XRF method) was 7.7 wt.%, and, after processing in a thermal plasma stream with enthalpy of 2.4 kW∙h/m^3^, the concentration of cobalt decreased to 4.6 wt.%. The treatment of granules in plasma at an enthalpy of 4.9 kW∙h/m^3^ reduced cobalt content in powder to 3.7 wt.% (Table 2).

When processing microgranules in a plasma flow, the carbon content in them also decreases. If the initial carbon content in granules before plasma treatment was 6.4 wt.%, after treatment in the plasma flow with enthalpy 2.4 kW∙h/m^3^, the carbon content decreased to 4.7 wt.% and for enthalpy 4.9 kW∙h/m^3^ to 3.9 wt.% (Table 2). Carbon in microgranules is present in the form of tungsten carbide phases, in a free state, and in PVB, which was used as a binder in the production of microgranules. The synthesized nanopowder had a carbon content of 4.7 wt.%, which suggests that when the microgranules were treated in a plasma flow, the carbon carryover was mainly due to the formation of gaseous carbon compounds during pyrolysis of PVB. Thus, the carbon introduced into the microgranules by the binder should not be accounted for in the carbon balance, which is involved in the chemical transformation of tungsten carbide phases during treatment/spheroidization of nanopowder microgranules in a plasma stream.

The oxygen content in the spheroidized powder was at the level of 0.03–0.05 wt.% and was determined by the intensity of initial granules interaction with plasma flow and its enthalpy level. The radical decrease in oxygen in powder from 1.0 wt.% in granules to 0.03 wt.% in spheroidized powder is determined first of all by the reductive chemical reactions of hydrogen with oxygen-containing granules components. The contribution of reactions in which the carbon of the W-C-Co composition and the carbon-containing products of thermal decomposition of the organic binder cannot be excluded.

## 4. Conclusions

The performed set of experimental studies has shown the principal possibility of obtaining dense spherical microparticles based on the composition of tungsten carbides and cobalt having a submicron structure by the consecutive use of plasma-chemical synthesis of a W-C-Co system nanopowder, spray drying of suspension based on a W-C-Co system nanopowder with obtaining nanopowder microgranules and spheroidization of microgranules in a thermal plasma flow.

It is found that the treatment of nanopowder microgranules in a thermal plasma stream leads to a change in their chemical composition: a reduction of carbon, oxygen, and cobalt, and the formation of nano- and submicron particles. To eliminate these negative effects, it is necessary to carry out further research aimed at investigating the development of methods to control the structure and chemical and phase composition of the obtained spherical microparticles of the tungsten carbide-cobalt system at the various stages in the process of their production in order to optimize their properties for the use of the micropowders in the manufacture of hard metal products by methods of additive technologies.

The study was supported by a grant from the Russian Science Foundation (project No. 19–73-00275).

## Figures and Tables

**Table 1:** Characteristics of W-C-Co powders.

	Microgranules	Spheroidized Powder Produced at Different Enthalpy	
		2.8 kW∙h/m^3^	4.8 kW∙h/m^3^
D_av_, μm	39.1	16.7	19.8
Apparent density, g/cm^3^	2.6	8.8	9.5
Flowability, s/50 g	29	11	10

**Table 2:** Composition of powders obtained.

	Nanopowder	Microgranules	Spheroidized Powder Produced at Different Enthalpy	
			2.8 kW∙h/m^3^	4.9 kW∙h/m^3^
Carbon, wt.%	4.7	6.4	4.7	3.9
Cobalt, wt.%	7.7	7.0	4.6	3.7
Oxygen, wt.%	0.5	1.0	0.05	0.03

## Data Availability

Not applicable.
